# Notification of Disease

**Published:** 1904-10-08

**Authors:** 


### The Hospital.

A JOURNAL OF

**The Medical Sciences and Hospital Administration.**

VOL. XXXVII.—No. 941. OCTOBER 8, 1904.

### NOTIFICATION OF DISEASE.

THE account of its stewardship for 1903-1904, just rendered by the Local Government Board, deals with so astounding a multiplicity of subjects, many of which are of great practical importance, and all of which are disposed of within short compass, that it is difficult to select from among them those which are most worthy of comment, or concerning which the materials furnished are sufficiently abundant to justify it. We feel no difficulty, however, in giving the first place to the statistics of infectious disease, which at least serve to call attention to the prodigious magnitude of the question to which they relate, and which appear to afford a far more accurate test of the general condition of the public health than can ever be furnished by returns of mortality alone. The statistics in question would seem to belong more properly to the report of the Medical Officer of the Board than to the general one of departmental work; and, if they could in future be transferred to the former, they would doubtless be made subjects of enlightening comment with regard to the distribution and the causes of the diseases to which they relate. They do not cover the whole of England and Wales, but relate only to 246 urban sanitary districts, with an aggregate population of rather more than nineteen millions (19,043,130) out of the total estimated population of 32,997,626; and, for some reason which is not stated, they do not include several diseases which are at once extremely prevalent and causes of large mortality. Thus, they make no mention of measles, by which, in 1902, 12,930 deaths were occasioned, 93 per cent. of them in children under five years of age, nor of whooping-cough, by which 9,805 deaths were occasioned in 1902, 97 per cent. of them in children under five years of age, nor of the diarrhœal group; and only include small-pox, scarlet fever, diphtheria and croup, enteric and other "fevers," cholera, erysipelas, and plague—a trio concerning two of which the history is happily negative. Still, imperfect as they are, they show the occurrence during the year to which they relate, and in the limited population of only 19 millions, of no less than 155,201 cases of infectious disease, and of 10,299 deaths from them. If we estimate the cost of an illness, direct and indirect, at only £1, we obtain a total loss to the country, in little more than half its population, of an amount which would capitalise at over £5,000,000 sterling, and which it would be cheap to avoid at that amount of expenditure.

The statistics given appear to relate to the calendar year 1903; and they show, for the nineteen millions, 12,586 cases of "enteric or typhoid" fever, with 2,081 deaths. It would seem likely, at first sight, that the proportionate prevalence of this disease would be greater in the urban sanitary districts than in the rural districts, for which the figures are not given, but further investigation seems to show that this was not the case. In the year 1902, according to the report of the Registrar-General, the deaths from enteric fever of 4,149 persons were registered in England and Wales. The numbers may not have been identical in the two successive years, but, assuming them to have been nearly so, the urban districts have scarcely more than half the total mortality in, roughly, 60 per cent. of the total population, and the rural districts have little less than half the total mortality in the remaining 40 per cent. The same applies to diphtheria and croup, from which the cases and deaths of 1903 were respectively 37,345 and 3,708 among the urban 60 per cent. of population, while the deaths of 1902 were 8,411 among the entire population, thus leaving 4,703 for the rural 40 per cent. In scarlet fever, on the contrary, the balance seems to have been more even. The cases among the 60 per cent. of population were 78,168, and the deaths 2,693; while the total deaths of 1902 were 4,875, leaving 2,182 for the 40 per cent. With regard to some of the notifiable diseases, and especially with regard to enteric fever, we are told by the Registrar-General that it was much more fatal in towns than in the country; and if this be so, we can only suppose that the comparative prevalence must have been much greater in the rural districts, in order to produce an absolutely larger number of deaths in a smaller population. It is well known that at the time when the late Dr. William Budd instituted his classical researches into the causes and diffusion of enteric fever it was mainly a disease of rural districts, spreading from village to village along the tracks of brooks and watercourses, and it is quite possible that the increased protection of water from excremental pollution, which has presumably been the chief cause of a diminished incidence of the disease upon urban populations, has not been operative to the same extent in rural districts. Whatever may be the explanation, the facts seem to call for the very careful consideration of rural sanitary authorities, and of the medical officers of health who advise them. That there should be 2,081 deaths from enteric fever among 19 millions of urban population, against 2,068 among 13 millions of rural population is a fact which calls loudly for explanation, and there can be little doubt that the explanation when afforded would throw much light upon the general question of the prevalence of the disease. The great diminution in enteric fever mortality which has been produced during the latter half of the last century and the diminution in the mortality from scarlet fever, which is now described by Dr. Tatham as "insignificant compared with what it was even so recently as 30 years ago," are not matters upon which we should dwell with a contentment antagonistic to continued effort, or with any reasonable expectation that the improvement will be maintained. The fact that it has been possible greatly to diminish the prevalence or the fatality of these or other diseases points to the possibility of extinguishing or of curing them. There must have been at least 25,000 cases of enteric fever in the country last year, and their occurrence can only be regarded as a serious indictment either of our sanitary laws or of their administration.

